# Influence of upper anterior teeth crowding on the quality of life of children and adolescents

**DOI:** 10.1007/s00056-025-00595-w

**Published:** 2025-08-19

**Authors:** Alice von Laffert, Sandra Riemekasten, Wieland Kieß, Antje Körner, Till Koehne, Christian Hirsch

**Affiliations:** 1 Department of Orthodontics, University Leipzig, Liebigstr. 12, 04103 Leipzig, Germany; 2 Department of Women and Child Health, Hospital for Children and Adolescents and Center for Pediatric Research (CPL), University of Leipzig, Liebigstr. 20a, 04103 Leipzig, Germany; 3 Department of Paediatric and Preventive Dentistry, University Leipzig, Liebigstr. 12, 04103 Leipzig, Germany; 4 LIFE Leipzig Research Center for Civilization Diseases, University of Leipzig, Philipp-Rosenthal-Str. 27, 04103 Leipzig, Germany

**Keywords:** Social well-being, Emotional well-being, Orthodontic treatment need, Oral health-related quality of life, Tooth malocclusions, Orthodontics, Soziales Wohlbefinden, Emotionales Wohlbefinden, Kieferorthopädischer Behandlungsbedarf, Mundgesundheitsbezogene Lebensqualität, Zahnfehlstellungen, Kieferorthopädie

## Abstract

**Purpose:**

The indication for orthodontic treatment in children and adolescents is based on severity of tooth and jaw malocclusions that lead to limitations in oral health-related quality of life (QoL). We have already shown how overbite and overjet deviations negatively affect QoL. However, few studies have investigated the influence of anterior crowding in the upper jaw on QoL.

**Methods:**

We selected 734 children and adolescents aged 11–14 years from a population-based longitudinal cohort study (LIFE Child). The data used in this study were retrospectively analyzed. Evaluable jaw models were available for 554 subjects in which we performed three orthodontic space analyses: (i) purely visual assessment of the crowding of the incisors in the upper jaw (VA), (ii) the Little index (LI), and (iii) the Lundström analysis (LA). Limitations in QoL were determined using the German version of the Child Perceptions Questionnaire (CPQ-G-11-14), taking into account the effects of age, gender, socioeconomic status (Winkler score), orthodontic treatment, oral hygiene, overbite/overjet, and dental caries (decayed, missing, filled teeth [DMFT] index) as possible influencing factors.

**Results:**

The percentage of individuals with crowding in our cohort was 11% according to VA, 14.3% according to LI, and 7.3% according to the LA. Within the three analyses, the groups of individuals with crowding did not fully overlap. The CPQ score (subscales: “emotional and social well-being”) was significantly higher in subjects with crowding independent of the type of crowding analysis (*p* < 0.05), gender, or socioeconomic status (multivariable linear regression analysis).

**Conclusion:**

Children and adolescents with crowding had significantly more QoL impairments, especially related to emotional and social well-being. It is necessary to perform more than one crowding analysis in order to find more affected individuals with a reduced QoL due to crowding.

## Abstract (de)

**Zielsetzung:**

Die Indikation für eine kieferorthopädische Behandlung bei Kindern und Jugendlichen richtet sich nach dem Schweregrad der Zahn- und Kieferfehlstellungen, die zu Einschränkungen der mundgesundheitsbezogenen Lebensqualität (QoL) führen. Wir haben bereits gezeigt, wie ein abweichender Overjet- und Overbite die QoL negativ beeinflussen. Es gibt jedoch nur wenige Studien, die den Einfluss eines frontalen Engstandes im Oberkiefer auf die QoL untersucht haben.

**Methoden:**

734 Kinder und Jugendliche im Alter von 11–14 Jahren wurden aus einer bevölkerungsbasierten Längsschnitt-Kohortenstudie (LIFE Child) ausgewählt. Die in dieser Studie verwendeten Daten wurden retrospektiv ausgewertet. Für 554 Probanden standen auswertbare Kiefermodelle zur Verfügung, an denen wir 3 Analysen zum kieferorthopädischen Platzbedarf durchführten: (i) eine rein visuelle Beurteilung des Engstandes der oberen Inzisiven (VA), (ii) den Little-Index (LI) und (iii) die Lundström-Analyse (LA). Einschränkungen der QoL wurden mit der deutschen Version des Child Perceptions Questionnaire (CPQ-G-11-14) ermittelt, wobei die Auswirkungen von Alter, Geschlecht, sozioökonomischem Status (Winkler-Score), kieferorthopädischer Behandlung, Mundhygiene, Overbite/Overjet und Karies (Index kariöser, fehlender, gefüllter Zähne [DMFT]) als mögliche Einflussfaktoren berücksichtigt wurden.

**Ergebnisse:**

Der prozentuale Anteil der Personen mit Engstand in unserer Kohorte betrug 11 % nach VA, 14,3 % nach LI und 7,3 % nach LA. Innerhalb der 3 Analysen überschnitten sich die Personengruppen mit Engstand nicht vollständig. Der CPQ-Score (Subskalen: „emotionales und soziales Wohlbefinden“) war bei Personen mit Engstand signifikant höher, unabhängig von der Art der Engstandsanalyse (*p* < 0,05), dem Geschlecht oder dem sozioökonomischen Status (multivariable lineare Regressionsanalyse).

**Schlussfolgerung:**

Kinder und Jugendliche mit Engstand hatten signifikant mehr Beeinträchtigungen der Lebensqualität, insbesondere in Bezug auf ihr emotionales und soziales Wohlbefinden. Um mehr Betroffene mit einer reduzierten LQ aufgrund von Engstand zu identifizieren, ist es notwendig, mehr als eine Engstandsanalyse durchzuführen.

## Introduction

Healthy self-esteem is important for children’s development and their mental and physical health [1]. Therefore, individuals’ quality of life should be considered when assessing the need for orthodontic treatment. Part of overall health-related quality of life is oral health [2], which refers specifically to the condition of the teeth and jaws.

Tooth malocclusions can affect patient’s appearance and therefore their self-confidence. In fact, we and others have already shown that malocclusion and jaw malposition have an impact on oral health-related quality of life (QoL) in children and adolescents [3–7]. To measure impairments in oral health-related QoL, Bekes et al. [8] developed a questionnaire that primarily describes the self-perception of a patient’s oral health and its impact on the social environment.

Individuals’ responses to these questions provide information on the impact of oral health-related QoL on daily life, general health, and psychological status.

Tooth crowding of the upper anterior teeth inevitably leads to esthetic impairment, as the lack of space leads to rotation and tilting of the teeth. Studies have already shown that crowded anterior teeth negatively affect the quality of life of young adults and adolescents [9, 10]. However, these studies only used a clinical assessment of crowding using the Dental Aesthetic Index, whereas quantitative orthodontic model analysis is still the gold standard for identifying individuals with crowding. Accordingly, in a systematic review and meta-analysis, Göranson et al. [11] concluded that many studies investigating malocclusions and its influence on the quality of life in children and adolescents were of moderate quality. Therefore, it is necessary to use standardized measuring methods to estimate crowding of the teeth and a standardized questionnaire to evaluate QoL in future studies.

It was hypothesized in this study that upper anterior crowding negatively impacts oral health-related quality of life (QoL) in children and adolescents, as determined by validated analyses of jaw models and QoL questionnaires. Specifically, it was expected that higher crowding scores from the analyses of three jaw models would correlate with greater constraints on QoL, as measured by reliable instruments for this population.

## Individuals and methods

### Subjects and data collection

The jaw models were obtained as part of a prospective population-based cohort study (LIFE Children’s Study as part of the “Leipzig Research Center for Civilization Diseases–LIFE”) in which more than 3500 children and adolescents up to the age of 18 were examined and interviewed. The participants were recruited in cooperation with various hospitals, kindergartens, and schools as well as through advertisements on television and press reports. Details have already been reported elsewhere [12, 13].

Before the study started, the children and parents were informed about the aim and procedure of the study and their written consent was obtained. As part of the examination program, a dental examination was carried out between May 2012 and April 2015. The dental examination was approved by the ethics committee of the University of Leipzig (Reg. No. 354-10-13122010).

### Evaluations and reliability

To assess the oral health-related QoL, the individuals completed the German version of the Child Perception Questionnaire (CPQ-G11-14), which was tested for reliability and validity in a population-based sample [8]. The original questionnaire was developed by a Canadian group led by Jokovic et al. [14] and contains 35 questions in four different categories: oral symptoms (5 questions), functional limitations (10 questions), emotional well-being (8 questions), and social well-being (12 questions). As reported by Bekes et al. [8], the German version of the CPQ11-14 was reliable and valid in the general population of 11- to 14-year-old German children.

The study persons filled out the questions on a computer using an online form. Their answers should reflect their feelings and observations during the last 3 months. The answers were classified as “never”, “once/twice”, “sometimes”, “often” and “every day/almost every day”. The individuals’ answers were linked to a score between 0 and 4. The sum of the scores varied between 0 and 140. A low score expresses good, stable mental health with few or no problems and a good to very good oral health-related quality of life. A higher score means that the patient’s quality of life is negatively affected by their oral health. The highest possible score of 140 points expresses a strong impairment of the oral health-related quality of life, meaning that problems occur “every day/almost every day”.

Before the clinical examination, the parents and the participants were asked about their monthly household income, their occupation, and their education to determine their socioeconomic status (Winkler index; [15]). In addition, whether or not orthodontic treatment took place was recorded by choosing between the option “yes” (orthodontic treatment with any type of appliance) or “no” (no orthodontic appliance).

We examined boys and girls between the ages of 11 and 14 years and correlated their crowding scores with their CPQ score in relation to age, gender, the Winkler index, oral hygiene, orthodontic treatment, overbite and overjet, and DMFT index.

During the first visit, a dental examination was performed, during which all relevant orthodontic measurements were taken, such as the classification of the bite and the measurement of the overbite and overjet. The DMFT index according to WHO criteria was collected from all subjects during the clinical examination. Oral hygiene was assessed using the Visible Plaque Index.

**Fig. 1:**
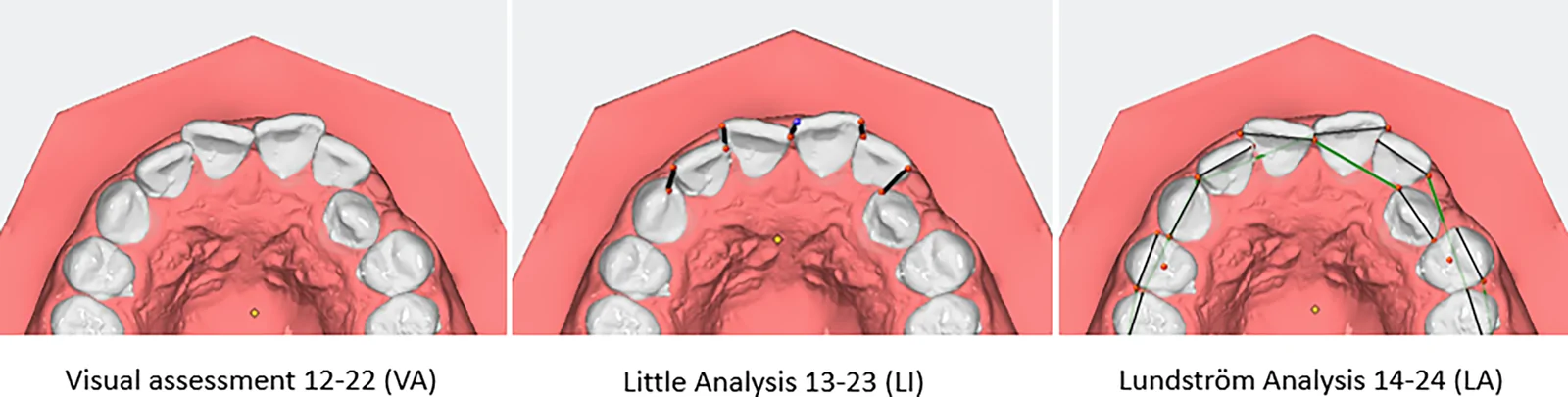
Depiction of how the three crowding analyses were performed: visual assessment, Little analysis, and Lundström analysis Darstellung der Durchführung der 3 Engstandsanalysen: visuelle Beurteilung, Little-Analyse und Lundström-Analyse

In addition, impressions of the upper and lower jaws including a bite registration was done by using the dual arch method and a A-silicon (®HS VPA Hydro Putty, Henry Schein Dental Deutschland GmbH, Langen, Germany). Standard plaster study models were produced and three different crowding analyses were carried out (Fig. 1).

At first, a visual assessment (VA) of the crowding of the four upper incisors was performed. Tooth crowding was defined according to Portillo et al. [16] as “mild” when the dental alveolar discrepancy was 0–3 mm, as “moderate” if it was > 3–5 mm, and “severe” if it was > 5 mm.

As a second model analysis of crowding, the Little index (LI) was recorded for all individuals using a caliper. Deviations of the incisal edges of the adjacent upper incisors were measured and summarized as previously described [17].

As a third analysis of crowding, the Lundström analysis (LA) was calculated using a caliper by measuring the mesiodistal tooth width of the two upper first premolars, the two upper canines, the two upper lateral incisors, and the two upper central incisors (defined as space required). In addition, a segmental analysis according to Lundström was carried out, measuring the width of the individual segments between the tooth contacts [18]. The segments were defined as S2 (measuring the width between the contact point between the upper right first premolar and the upper right second premolar and the contact point between the upper right canine and the upper right lateral incisor), S3 (measuring the width between the contact point between the upper right canine and the upper right lateral incisor and the contact point between the two upper central incisors), S4 (measurement of the width between the contact point of the two upper central incisors and the contact point of the upper left lateral incisor and the upper left canine), and S5 (measurement of the width between the contact point of the upper left lateral incisor and the upper left canine and the contact point of the upper left first premolar and the upper left second premolar) (defined as offered space). Then the difference between the space required and the space offered was calculated [18].

**Fig. 2:**
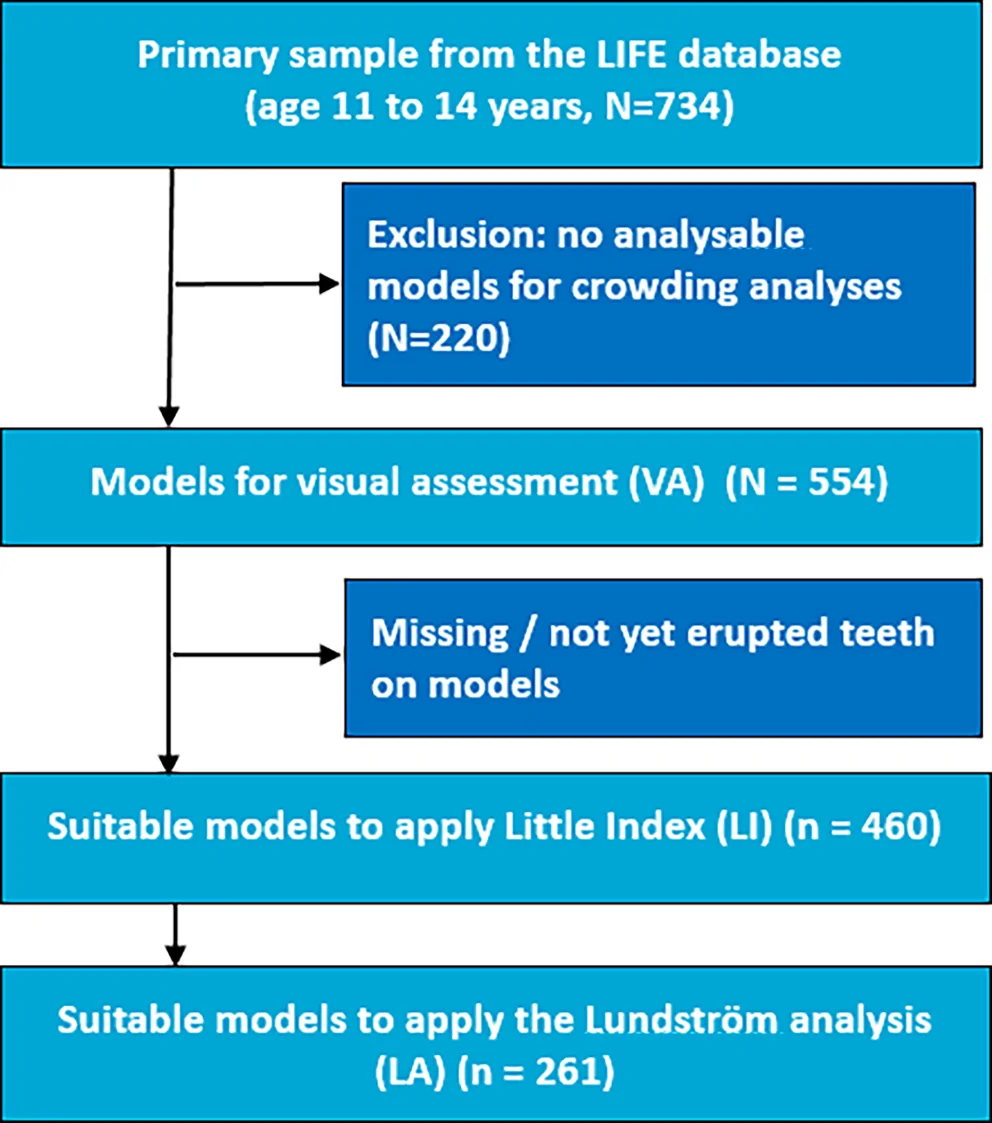
Flow diagram for identification of suitable models for visual assessment (VA), Little index (LI), and Lundström analysis (LA) Ablaufdiagramm zur Ermittlung geeigneter Modelle für die visuelle Beurteilung (VA), den Little-Index (LI) und die Lundström-Analyse (LA)

The visual assessment of crowding could be performed on all subjects as it is an assessment independent of the eruption status of the teeth. For the Little index, the presence of incisors and canines was mandatory, regardless of the state of the dentition. For the Lundström analysis, the presence of permanent teeth (first premolars, canines, and incisors) was mandatory (Fig. 2).

The evaluation of the jaw models and the dental examination is based on a standard operating procedure (SOP) that had to be learned in at least two training sessions by each of the four examiners (dentists and dental students under supervision).

In addition, we tested the interrater reproducibility of our method (dual arch impression) in comparison to the method used in practice (conventional alginate impression) for the fabrication of plaster models used for orthodontic model analysis. With an intraclass correlation (ICC) of > 0.85, the pretest showed very good agreement in the measurements (Table 1).

**Table 1:** Intraclass correlation (ICC) of models of manual alginate impressions (Alg. M) vs. models of manual dual arch impressions (DA M) Modelle manueller Alginatabformungen (Alg. M) vs. Modelle manueller Doppelkieferabformungen (DA M): Intraklassenkorrelation (ICC)

Alg. M vs. DA M	ICC
2nd molar	0.852
1st molar–1st premolar	0.997
Canine–incisor	0.986
*Lundström*	*0.996*
Transversal	0.998
Overbite	0.948
Overjet	0.881

### Statistical evaluation

First, we defined cut-off values for crowding in each analysis. In the clinical visual assessment (VA; classified an estimation of crowding by the dental–alveolar discrepancy) tooth crowding was defined as present when the dental–alveolar discrepancy was moderate or severe (> 3 mm) [16]. For the Little index (LI) of the maxilla, a cut-off for crowding was set here at > 5 mm according to the work of Antoszewka–Smith [19]. In the Lundström analysis (LA), the cut-off for crowding was set at > −2 mm based on clinical experience.

Second, the individuals with crowding were compared with those without crowding in each analysis with regard to various variables in bivariate analyses. The chi-square test was used for gender, orthodontic treatment, oral hygiene, and the Winkler index category. Age, overbite and overjet, DMFT, and CPQ‑G sum score and its domains (oral symptoms, functional limitations, emotional, and social well-being) were compared between individuals with and without crowding for all analyses (VA, LI, and LA) using the t‑test. All variables in our random population sample were tested for normal distribution.

Third, a multivariable linear regression analysis was performed to determine the extent to which the degree of crowding (VA, LI, LA) influences the OHRQoL. We have presented here the CPQ‑G score (95% confidence interval [CI]) for the domains ‘social and emotional well-being’, as we expected relevant effects of crowding on QoL in these two domains. The effects of gender and orthodontic treatment were controlled as potential confounders in these analyses. Other variables were not considered as there is no evidence in the literature that correlations between sociodemographic or clinical variables influence the relationship between OHRQoL impairments and crowding in this narrowly defined age group.

Finally, we used quantity diagrams to graphically illustrate the overlap between the various crowding analyses in our study population.

## Results

### Study population

**Fig. 3:**
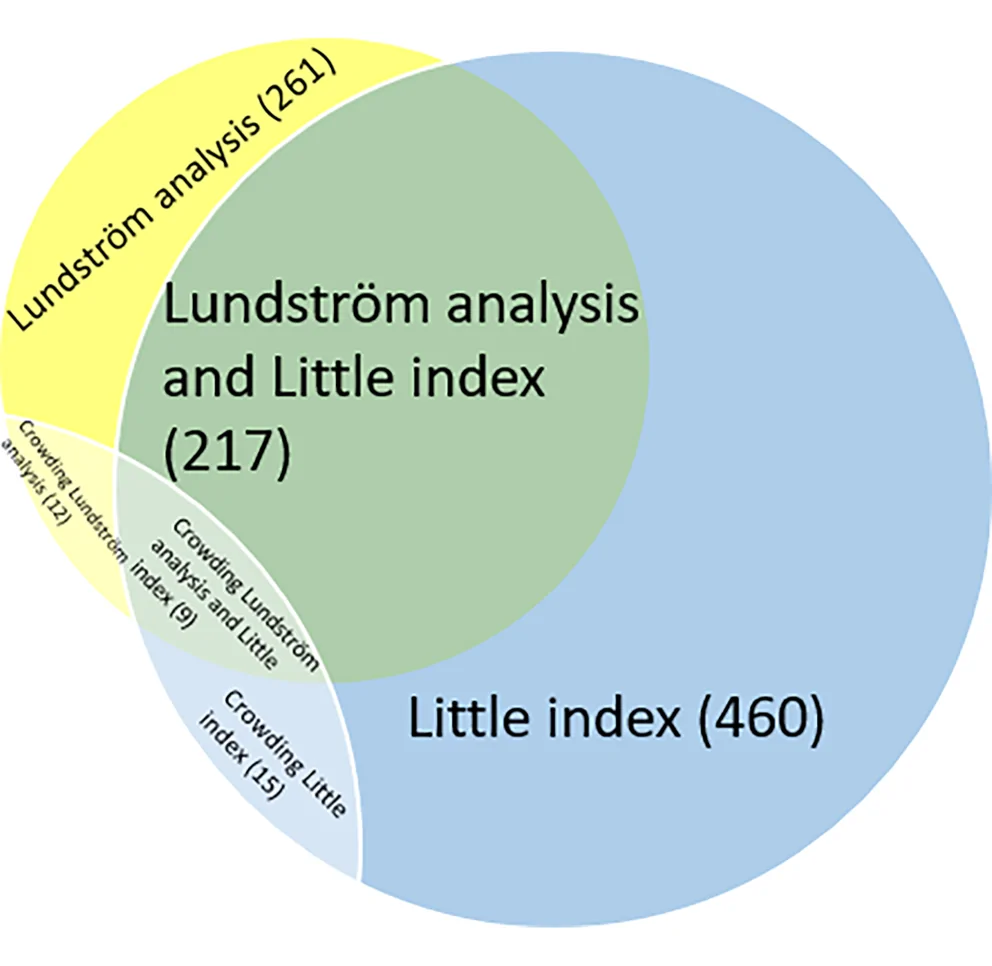
Overlap in crowding between Lundström analysis (LA) and Little index (LI) Überschneidungen zwischen Lundström-Analyse (LA) und Little-Index (LI) in Bezug auf den Engstand

**Fig. 4:**
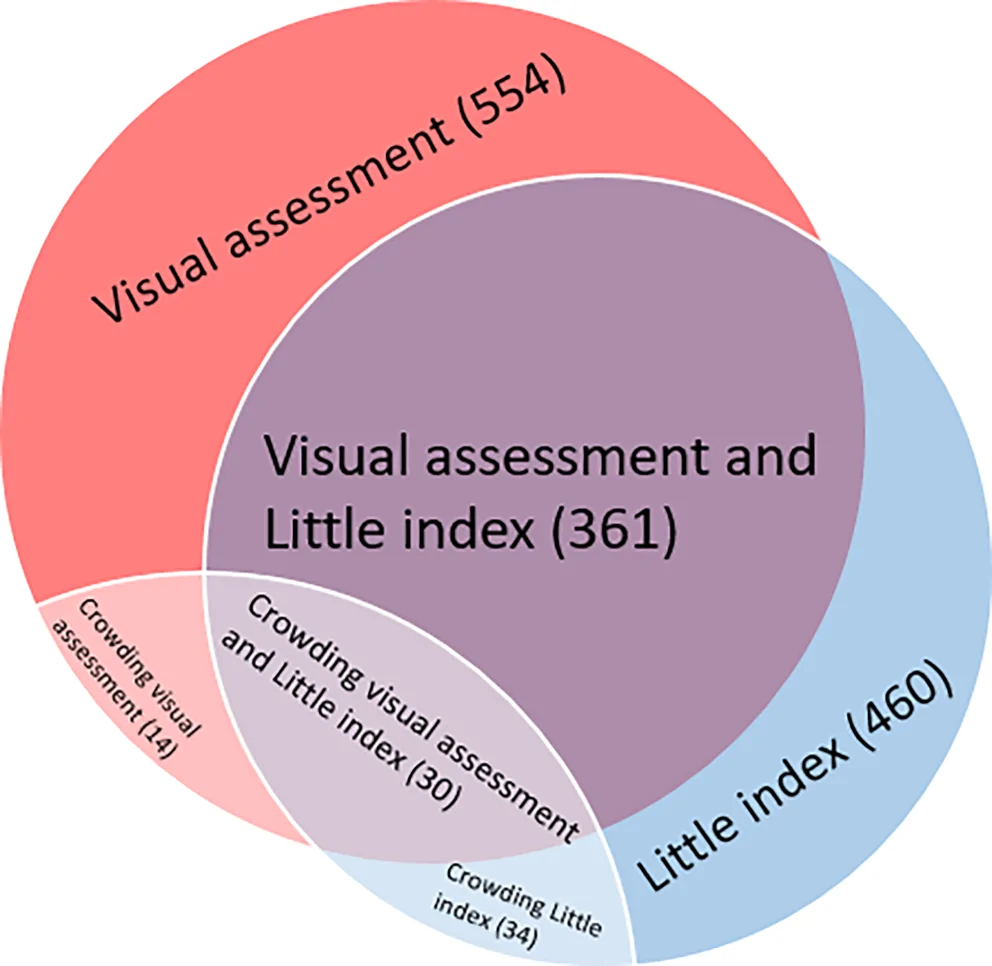
Overlap in crowding between Little index (LI) and visual assessment (VA) Überschneidungen zwischen Little-Index (LI) und visueller Beurteilung (VA) in Bezug auf den Engstand

**Fig. 5:**
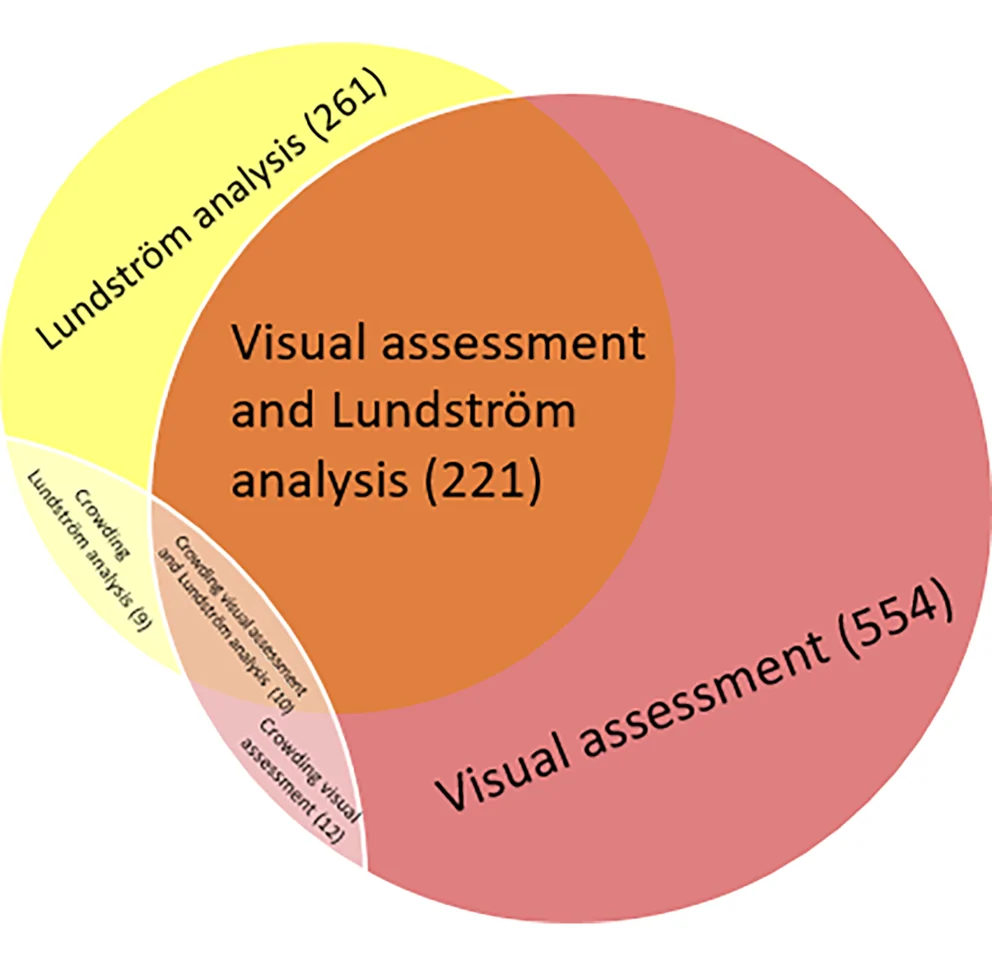
Overlap in crowding between visual assessment (VA) and Lundström analysis (LA) Überschneidungen zwischen visueller Beurteilung (VA) und Lundström-Analyse (LA) in Bezug auf den Engstand

When comparing the patients with crowding within the three model analyses, no complete congruence of the groups was recognizable, but a percentage distribution is observed (Figs. 3, 4, and 5).

The mean age of the study participants in the subsample of VA (*N* = 554) was 12.4 ± 1.4 years old, the mean age of the participants whose models were analyzed using LI (*N* = 460) were 12.6 ± 1.4 years old, and the mean age of the participants who received the LA (*N* = 261) were 13.3 ± 1.2 years old (Tables 2, 3, and 4).

**Table 2:** Comparison of subjects with crowding of the upper anterior teeth according to the visual assessment (> 1 width of a lower incisor) and those without in terms of sociodemographic and dental variables and oral health-related quality of life (OHRQoL) limitations Vergleich von Probanden mit Engstand der Oberkieferfrontzähne nach visueller Beurteilung (> eine Breite eines unteren Inzisivus) und Probanden ohne Engstand in Bezug auf soziodemographische und zahnmedizinische Variablen sowie Einschränkungen der mundgesundheitsbezogenen Lebensqualität (OHRQoL)

	Total VA *N* = 554	Crowding *N* = 54	No crowding *N* = 500	*P*^c^
*Sociodemographic*				
Age mean (SD)^b^	12.4 ± 1.4	12.2 ± 1.4	12.4 ± 1.4	n. s.
Female gender, % (*N*)^a^	273 (49%)	31 (57%)	242 (48%)	n. s.
SES middle, % (*N*)^3^	320 (58%)	31 (57%)	289 (58%)	n. s.
*Oral conditions*				
Orthodontic treatment, % (*N*)^a^	186 (34%)	13 (24%)	173 (35%)	n. s.
Bad oral hygiene, % (*N*)^a^	44 (8%)	6 (11%)	38 (8%)	n. s.
Overbite, mm mean (SD)^b^	3.6 ± 1.6	4.1 ± 2	3.5 ± 1.5	*0.01*
Overjet, mm mean (SD)^b^	3.1 ± 1.5	3.3 ± 2.0	3.1 ± 1.5	n. s.
DMFT, mean (SD)^b^	0.8 ± 1.6	0.6 ± 1.5	0.8 ± 1.6	n. s.
*OHRQoL*				
Summary score, mean (SD)^b^	8.2 ± 8.6	9.6 ± 12	8.1 ± 8.3	n. s.
Oral symptoms, mean (SD)^b^	2.8 ± 2.4	2.1 ± 1.7	2.8 ± 2.4	n. s.
Functional limitations, mean (SD)^b^	2.7 ± 3.2	2.1 ± 3.1	2.8 ± 3.2	n. s.
Emotional well-being, mean (SD)^b^	1.1 ± 2.6	2.5 ± 4.1	1.1 ± 2.4	*0.0006*
Social well-being, mean (SD)^b^	1.6 ± 3.4	2.9 ± 5.2	1.4 ± 3.2	*0.0058*

*SD* Standard deviation, *VA* Visual assessment, *SES* Socioeconomic status, *DMFT* Decayed, missing, filled teeth/all teeth

^a^Chi-square test

^b^t‑test

^c^statistically significant differences are shown in italics

**Table 3:** Comparison of subjects with crowding of the upper anterior teeth according to the Little index (> 5 mm between 13 and 23) and those without in terms of sociodemographic and dental variables and oral health-related quality of life (OHRQoL) limitations Vergleich von Probanden mit Engstand der Oberkieferfrontzähne nach dem Little-Index (> 5 mm zwischen 13 und 23) und Probanden ohne Engstand in Bezug auf soziodemographische und zahnmedizinische Variablen sowie Einschränkungen der mundgesundheitsbezogenen Lebensqualität (OHRQoL)

	Total LI *N* = 460	Crowding *N* = 66	No crowding *N* = 394	*P*^c^
*Sociodemographic*				
Age, mean (SD)^b^	12.6 ± 1.4	12 ± 1.5	12.7 ± 1.4	*0.000*
Female gender (*N*)^a^	235 (51%)	32	203	n. s.
SES middle, % (*N*)^3^	274 (63%)	39 (72%)	235 (47%)	n. s.
*Oral conditions*				
Orthodontic treatment, % (*N*)^a^	151 (33%)	15 (23%)	136 (35%)	n. s.
Bad oral hygiene, % (*N*)^a^	34 (7%)	6 (9%)	28 (7%)	n. s.
Overbite, mm mean (SD)^b^	3.6 ± 1.6	4.1 ± 1.8	3.5 ± 1.5	*0.016*
Overjet, mm mean (SD)^b^	3.1 ± 1.5	3.2 ± 1.6	3.1 ± 1.4	n. s.
DMFT, mean (SD)^b^	0.8 ± 1.7	0.4 ± 0.9	0.9 ± 1.7	*0.026*
*OHRQoL*				
Summary score, mean (SD)^b^	8.2 ± 8.7	9.7 ± 11.7	7.9 ± 8.2	n. s.
Oral symptoms, mean (SD)^b^	2.8 ± 2.4	2.4 ± 2.1	2.8 ± 2.4	n. s.
Functional limitations, mean (SD)^b^	2.7 ± 3.3	2.5 ± 3.5	2.8 ± 3.2	n. s.
Emotional well-being, mean (SD)^b^	1.1 ± 2.7	2.1 ± 3.8	1.0 ± 2.4	*0.0028*
Social well-being, mean (SD)^b^	1.5 ± 3.3	2.7 ± 5	1.3 ± 2.9	*0.0035*

*SD* Standard Deviation, *LI* Little index, *SES* Socioeconomic status, *DMFT* Decayed, missing, filled teeth/all teeth

^a^Chi-square test

^b^t‑test

^c^statistically significant differences are shown in italics

**Table 4:** Comparison of subjects with crowding of the upper anterior teeth according to the Lundström analysis (> −2 mm between 14 and 24) and those without in terms of sociodemographic and dental variables and oral health-related quality of life (OHRQoL) limitations Vergleich von Probanden mit Engstand der Oberkieferfrontzähne nach der Lundström-Analyse (> −2 mm zwischen 14 und 24) und Probanden ohne Engstand in Bezug auf soziodemographische und zahnmedizinische Variablen sowie Einschränkungen der mundgesundheitsbezogenen Lebensqualität (OHRQoL)

	Total (LA) *N* = 261	Crowding *N* = 19	No crowding *N* = 242	*p*^*c*^
*Sociodemographic*				
Age, mean (SD)^b^	13.2 ± 1.2	12.7 ± 1.1	13.3 ± 1.5	*0.0316*
Female gender, % (*N*)^a^	138 (53%)	11 (58%)	127 (52%)	n. s.
SES mean % (*N*)^3^	156 (60%)	10 (53%)	146 (60%)	n. s.
*Oral conditions*				
Orthodontic treatment, % (*N*)^a^	100 (38%)	6 (32%)	94 (39%)	n. s.
Bad oral hygiene, % (*N*)^a^	26 (10%)	6 (32%)	20 (8%)	*0.006*
Overbite, mm mean (SD)^b^	3.4 ± 1.6	2.8 ± 1.9	3.5 ± 1.5	*0.036*
Overjet, mm mean (SD)^b^	3.0 ± 1.6	2.6 ± 2.1	3.1 ± 1.6	n. s.
DMFT, mean (SD)^b^	1.0 ± 1.9	1.3 ± 1.9	1.0 ± 1.9	n. s.
*OHRQoL*				
Summary score, mean (SD)^b^	8.2 ± 8.9	11.9 ± 14.3	7.9 ± 8.4	n. s.
Oral symptoms, mean (SD)^b^	2.9 ± 2.4	3.1 ± 2.8	2.9 ± 2.3	n. s.
Functional limitations, mean (SD)^b^	2.7 ± 3.2	3.1 ± 4.3	2.7 ± 3.1	n. s.
Emotional well-being, mean (SD)^b^	1.2 ± 2.7	2.4 ± 4.4	1.1 ± 2.5	*0.047*
Social well-being, mean (SD)^b^	1.4 ± 3.3	3.3 ± 6.6	1.3 ± 2.9	*0.015*

*SD* Standard Deviation, *LA* Lundström analysis, *SES* Socioeconomic status, *DMFT* Decayed, missing, filled teeth/all teeth

^a^Chi-square test

^b^t‑test

^c^statistically significant differences are shown in italics

When the VA was performed, we identified 54 (11%) of 554 individuals with crowding using a cut-off of 1 width of an average lower incisor. Individuals with crowding had a significantly higher CPQ-sum score and a positive correlation with a reduced emotional and social well-being (*p* = 0.0006, *p* = 0.0058; Table 2).

When the LI was performed, we identified 66 (14%) of 460 individuals with crowding using a cut-off of 5 mm. Individuals with crowding had a significantly higher CPQ-sum score and a positive correlation with a reduced emotional and social well-being (*p* = 0.0028, *p* = 0.0035; Table 3). Interestingly, increasing age correlated with a decrease in crowding (*p* < 0.000) according to LI. Participants with crowding had higher overbite values (*p* = 0.016). There was no correlation with gender, SES, poor oral hygiene, orthodontic treatment or the overjet (Table 3).

When the LA was performed, we identified 19 (7.3%) of 261 individuals with crowding using a cut-off of −2 mm. Individuals with crowding according to LA had a significantly higher CPQ-sum score and a positive correlation with a reduced emotional and social well-being (*p* = 0.047 and *p* = 0.015; Table 4). Increasing age correlated with a decrease in crowding (*p* = 0.0316; Table 4). There was no correlation with gender and SES. However, there was a positive correlation of individuals with crowding and poor oral hygiene (*p* = 0.006; Table 4).

The multivariate analysis revealed a reduced emotional and social well-being in patients with crowding in all three analyses independently of gender (Tables 5 and 6). The impairments were reflected in an increase in the respective scale values by 1.09 to 2.05 units. This corresponded to an approximate doubling of the problem reports. Orthodontic treatment seemed to alleviate this impairment somewhat in the LA subsample.

**Table 5:** Multivariate analysis regarding emotional well-being in all three crowding analyses Multivariate Analyse zum emotionalen Wohlbefinden in allen 3 Engstandsanalysen

	Coefficient	95% CI	*P*^a^
Variables	Emotional well-being		
*Crowding according to VA (>* *1 IW)*	*1.36*	*0.60–2.17*	*0.001*
Sex (female)	0.09	−0.38 to 0.55	n. s.
Orthodontic treatment	−0.07	−0.26 to 1.74	n. s.
*Crowding according to LI (>* *5* *mm)*	*1.09*	*0.34–1.83*	*0.004*
Sex (female)	0.32	−0.19 to 0.84	n. s.
Orthodontic treatment	−0.12	−0.33 to 0.10	n. s.
*Crowding according to LA (<* *−2* *mm)*	*1.32*	*0.0–2.67*	*0.05*
Sex (female)	0.21	−0.48 to 0.90	n. s.
Orthodontic treatment	−0.28	−0.56 to 0.00	0.048

*IW* Incisor width, *VA* Visual assessment, *LI* Little index, *LA* Lundström analysis, *95% CI* 95% confidence interval, *n.s.* not significant

^a^statistically significant differences are shown in italics

**Table 6:** Multivariate analysis regarding social well-being in all three crowding analyses Multivariate Analyse zum sozialen Wohlbefinden in allen 3 Engstandsanalysen

	Coefficient	95% CI	*P*^c^
Variables	Social well-being		
*Crowding according to VA (>* *1 IW)*	*1.44*	*0.4–2.48*	*0.007*
Sex (female)	−0.49	−0.09 to 0.11	n. s.
Orthodontic treatment	−0.21	−0.46 to 0.04	n. s.
*Crowding according to LI (>* *5* *mm)*	*1.25*	*0.33–2.18*	*0.009*
Sex (female)	−0.4	−1.03 to 0.24	n. s.
Orthodontic treatment	−0.23	−0.5 to 0.04	n. s.
*Crowding according to LA (<* *−2* *mm)*	*2.05*	*0.42–3.68*	*0.014*
Sex (female)	−0.37	−1.21 to 0.47	n. s.
Orthodontic treatment	−0.34	−0.67 to 0.00	0.047

*IW* Incisor width, *VA* Visual assessment, *LI* Little index, *LA* Lundström analysis, *95% CI* 95% confidence interval, *n.s.* not significant

^c^statistically significant differences are shown in italics

When comparing individuals with crowding between the three model analyses, we observed only a relatively small overlap between the three analyses (Figs. 3, 4, and 5). In particular, while 36 of 217 individuals showed crowding in either the LA or LI analyses, only 9 of these 36 individuals (25%) showed crowding in both analyses simultaneously (Fig. 3). Comparing the VA and LI analyses (Fig. 4), 30 of 78 individuals (38%) with crowding in one analysis also had crowding in both analyses. Comparing the VA and LA analyses (Fig. 5), 10 of 31 individuals (32%) with crowding in one analysis also had crowding in both analyses.

## Discussion

All three crowding analyses showed a significant correlation between the presence of major crowding and quality of life. In particular, the “emotional well-being” and “social well-being” domains were reduced in children and adolescents with crowding, which may indicate psychological problems associated with crowded teeth. In line with this finding, Gupta et al. [20] reported that dental malocclusions in general (protrusion, crowding, spacing, increased overbite) negatively affected the emotional well-being of children. This study also found that defects such as caries, enamel hypoplasia or damaged teeth following trauma negatively affected children’s mental health [20]. An increased overbite (> 6 mm) also reduced quality of life [3]. In this study, the “oral symptoms” and “functional limitations” domains of the oral health-related QoL were particularly affected by an increased overjet > 6 mm. Rusanen et al. [21] also showed in their study that malocclusions can cause physical and psychological discomfort and negatively affect self-confidence. It is well known that children and adolescents need stable self-esteem during adolescence in order to develop a healthy psyche. Nowadays, comparing and competing with other peers is becoming more common as social media provides a great platform for self-expression and showing off one’s image. The use of “filters” to enhance face, skin, and body shape is widespread and leads to distorted perceptions. Goldstein et al. [22] found that the eyes and mouth contribute most to a person’s attractiveness, which includes a harmonious smile. As the use of social media leads to higher standards of appearance, children and adolescents may be more sensitive to small imperfections such as rotations, tippings, or other types of malocclusion in the anterior region due to crowding.

Crowded teeth did not cause or affect the areas of oral health-related QoL “functional limitations”, “pain”, or other “oral symptoms”. This proves that a slight misalignment of the teeth in the area of the first premolars and anterior teeth did not have a major impact on malocclusion that could lead to more severe functional problems or even pain.

An interesting finding of this study was that the effect of crowded teeth on oral health-related QoL was independent of gender and orthodontic treatment. This result refutes the common theory that girls are more concerned about their appearance than boys.

Another important finding of this study was that only one analysis to detect crowding in individuals may not find all affected individuals. It is therefore necessary to perform several analyses to detect all individuals with crowding and the resulting reduced quality of life. The reason for the incomplete congruence of individuals with crowding in the different model analyses remains unclear. One explanation could be that fewer/more teeth and/or the weighting of the individual deviations of the teeth from the dental arch within the individual analyses. For example, the Little index calculates the sum of the deviations, whereas the visual assessment compares the individual deviations of the teeth from the dental arch (in incisor widths).

In some European countries, the severity of dental malocclusions determines whether treatment is financed by the general health insurance or not. The severity is classified using the Index of Orthodontic Treatment Need (IOTN) system or its national adaptations (e.g., KIG system in Germany) [23]. In these systems, the severity of the malocclusion is assessed in terms of sagittal, transversal, and vertical deviations [24]. Other criteria include the absence of permanent teeth, the retention of teeth, craniofacial anomalies as well as crowding and lack of space [24]. Precise measurements on the patient’s dental model determine the severity of malocclusion and the classification into the different categories. The development of this system was based on epidemiological studies and expert judgement; the patient’s own perception is not taken into account. The aim of the classification is to enable sensible, cost-effective orthodontic treatment and, thus, to prevent supposedly unnecessary treatments that are carried out for purely esthetic reasons [25] from being financed by the statutory health insurance. This means that only treatments that improve the patient’s functional limitation and tooth preservation should be funded. In the German classification system, orthodontic treatment for crowding is only covered if an anterior tooth deviates more than 3 mm from the ideal dental arch, and for the canines and the premolars there needs to be a loss of space of more than 3 mm for aligning these teeth, comparable with IOTN grade 3. The missing space is measured by creating an ideal dental arch and measuring the distance between the contact points of the adjacent teeth (anterior teeth) or the missing space in the ideal dental arch of the gap for the placement of the crowded tooth (canines and premolars). Since this study found that even 2 mm of crowding in the LA and > 5 mm of crowding in the LI had an impact on the emotional well-being of children and adolescents (measured across the dental arch from the right to the left first premolar), it can be suggested that funding and implementing orthodontic treatment may be appropriate even for less pronounced crowding than proposed in the KIG classification (or international IOTN). Reduced emotional well-being could lead to further psychological problems, which could result in even higher costs for the health care system. While the classification of malocclusions can be helpful in determining the need for orthodontic treatment, personal perception and the influence of reduced dental esthetics should also be taken into account when deciding on the need for orthodontic treatment. In fact, Demirovic et al. [26] were able to show that the quality of life increased after orthodontic treatment within their patient cohort. Importantly, theses authors used the same questionnaire regarding the measurement of quality of life as that used in our study.

Since all three crowding analyses (two model analyses and a visual assessment) gave similar results in terms of correlation with emotional and social QoL, it can be concluded that the practitioner has a high probability of filtering out individuals with crowding that negatively affects QoL using multiple crowding analyses. The initial clinical impression of the orthodontist or dentist can be supported by a subsequent model analysis and the diagnosis can be reliably derived from it in order to initiate appropriate orthodontic treatment.

There are some limitations in our study since participation in the study was dependent on the decision and willingness of the parents, which in turn has made access to the study difficult or even impossible for children from disadvantaged backgrounds. This is a unicentric study that assumes participants live close to the clinic, so further multicentric studies could represent a larger cross-section by analyzing larger and different areas.

In conclusion, also individuals with tooth crowding that does not require treatment (according to the IOTN system) could benefit from an orthodontic treatment and a proper teeth alignment.

## Data Availability

Data is provided within the manuscript.

## Abbreviations

Alg. M: Models of manual alginate impressions
CpQ: Child perceptions questionnaire
DA M: Models of manual dual arch impressions
DMFT: Decayed, missing, filled teeth
IOTN: International orthodontic treatment need
IW: Incisor width
KIG: *Kieferorthopädische Indikationsgruppe* (Orthodontic indication group)
LA: Lundström analysis
LI: Little index
LIFE: Leipzig Research Center for Civilization Diseases
OHRQoL: Oral health-related quality of life
QoL: Quality of life
SES: Socioeconomic status
SOP: Standard operating procedure
VA: Visual assessment
